# Dynamic hyperinflation is associated with a poor cardiovascular response to exercise in COPD patients

**DOI:** 10.1186/1465-9921-12-150

**Published:** 2011-11-10

**Authors:** Panagiota Tzani, Marina Aiello, Davide Elia, Luca Boracchia, Emilio Marangio, Dario Olivieri, Enrico Clini, Alfredo Chetta

**Affiliations:** 1 Cardiopulmonary Dept, Lung Function Unit, University Hospital (via Rasori 10), Parma (43125), Italy; 2 Cardiopulmonary Dept, Respiratory Disease Unit, University Hospital (via Rasori 10), Parma (43125), Italy; 3 Department of Oncology, Haematology, Respiratory Diseases and Ospedale Villa Pineta, (Via Gaiato 127), Pavullo (MO) (41026), University of Modena-Reggio Emilia, Modena, Italy

**Keywords:** dynamic hyperinflation, cardiovascular response, exercise, COPD

## Abstract

**Background:**

Pulmonary hyperinflation has the potential for significant adverse effects on cardiovascular function in COPD. The aim of this study was to investigate the relationship between dynamic hyperinflation and cardiovascular response to maximal exercise in COPD patients.

**Methods:**

We studied 48 patients (16F; age 68 yrs ± 8; BMI 26 ± 4) with COPD. All patients performed spirometry, plethysmography, lung diffusion capacity for carbon monoxide (TLco) measurement, and symptom-limited cardiopulmonary exercise test (CPET). The end-expiratory lung volume (EELV) was evaluated during the CPET. Cardiovascular response was assessed by change during exercise in oxygen pulse (ΔO_2_Pulse) and double product, i.e. the product of systolic blood pressure and heart rate (DP reserve), and by the oxygen uptake efficiency slope (OUES), i.e. the relation between oxygen uptake and ventilation.

**Results:**

Patients with a peak exercise EELV (%TLC) ≥ 75% had a significantly lower resting FEV_1_/VC, FEF_50_/FIF_50 _ratio and IC/TLC ratio, when compared to patients with a peak exercise EELV (%TLC) < 75%. Dynamic hyperinflation was strictly associated to a poor cardiovascular response to exercise: EELV (%TLC) showed a negative correlation with ΔO_2_Pulse (*r = - 0.476, p = 0.001*), OUES (*r = - 0.452, p = 0.001*) and DP reserve (*r = - 0.425, p = 0.004*). Furthermore, according to the ROC curve method, ΔO_2_Pulse and DP reserve cut-off points which maximized sensitivity and specificity, with respect to a EELV (% TLC) value ≥ 75% as a threshold value, were ≤ 5.5 mL/bpm (0.640 sensitivity and 0.696 specificity) and ≤ 10,000 Hg · bpm (0.720 sensitivity and 0.783 specificity), respectively.

**Conclusion:**

The present study shows that COPD patients with dynamic hyperinflation have a poor cardiovascular response to exercise. This finding supports the view that in COPD patients, dynamic hyperinflation may affect exercise performance not only by affecting ventilation, but also cardiac function.

## Introduction

Patients with COPD may develop pulmonary static hyperinflation because of destruction of pulmonary parenchyma and loss of elastic recoil. In addition to or independently from static hyperinflation, lung dynamic hyperinflation of the lung may be observed at any stage of COPD [1]. COPD patients with dynamic hyperinflation breathe in before achieving a full exhalation and, as consequence, air is trapped within the lungs with each further breath. Exertional dynamic hyperinflation implies a minor expansion of tidal volume and massive loading of inspiratory muscles with serious mechanical and sensory repercussions [2].

Pulmonary hyperinflation has the potential for significant adverse effects on cardiovascular function in COPD. In patients with severe emphysema, left and right ventricular performance was impaired because of small end-diastolic dimensions evaluated by magnetic resonance technique [3]. In these patients, decreased biventricular preload was attributed to intrathoracic hypovolemia caused by hyperinflated lungs. Futhermore in a population-based study, a greater extent of emphysema on CT scanning was related to impaired left ventricular filling, reduced stroke volume and lower cardiac output [4].

To our knowledge only one study [5] showed a relationship between dynamic hyperinflation and cardiovascular response in COPD patients performing a cycle ergometry (incremental test). In this study, the authors found that in patients with severe COPD, lower oxygen pulse was associated with resting and dynamic hyperinflation. Oxygen pulse (O_2_Pulse) is considered as a measure of cardiovascular efficiency, since it indicates what metabolic value in terms of oxygen uptake derives from every heart beat. It is commonly used as an estimator of stroke volume during exercise. However this remains controversial, especially in patients who desaturate [6]. It is of note that cardiovascular response to exercise may also be non invasively assessed by the double product (DP) [7], i.e. the product of systolic blood pressure and heart rate, and by the oxygen uptake efficiency slope (OUES) [8], i.e. the relation between oxygen uptake and ventilation. Thus, a battery of measures of cardiac function might provide a more comprehensive assessment of cardiovascular response to exercise.

The aim of this study was to investigate whether in COPD patients at any stage of disease, there is a relationship between dynamic hyperinflation and cardiovascular response to maximal exercise. Cardiovascular response was assessed by change during exercise in O_2_Pulse and in DP and by the OUES.

## Methods

### Subjects

We enrolled all consecutive patients suffering from COPD, defined according to GOLD criteria [9], referred for cardiopulmonary exercise testing (CPET) as part of a comprehensive functional evaluation between September 2010 and May 2011. All patients were smokers or ex smokers and they were on a stable optimized treatment for at least 1 month at the time of the study. Eligibility criteria were: 1) no clinical history of concomitant cardiac heart failure or anaemia 2) ability to perform a symptom-limited cycle ergometry cardio pulmonary test with a peak of respiratory exchange ratio (RER) ≥ 1.05 in order to exclude poor motivation 3) CPET stopped for fatigue and/or dyspnoea. All the procedures and their risks were explained to the patients, who gave their informed consent to enter the study. The pulmonary function testing as well as the exercise studies had been undertaken for clinical reasons at the request of the patient's clinician and the protocol was approved by the ethical review committee of the University Hospital of Parma.

#### Pulmonary function testing

Pulmonary function tests were performed according to international recommendations [10-12]. A flow-sensing spirometer and a body plethysmograph connected to a computer for data analysis (Vmax 22 and 6200, Sensor Medics, Yorba Linda, U.S.A.) were used for the measurements. Vital Capacity (VC), Forced Expiratory Volume at 1^st ^Second (FEV_1_), Forced Expiratory Flow measured at 50% of FVC (FEF_50 _in L/s) and Forced Inspiratory Flow measured at 50% of FVC (FIF_50 _in L/s) were recorded. FEV_1_/VC and FEF_50_/FIF_50 _ratios were taken as indices of airway obstruction and airway collapsibility, respectively.

Thoracic gas volume (TGV) was measured by body plethysmography with the subject panting against a closed shutter at a frequency slightly < 1 Hz and their cheeks supported by their hands. Total Lung Capacity (TLC) was obtained as the sum of TGV and linked Inspiratory Capacity (IC). IC/TLC was taken as an index of static hyperinflation of the lung.

At least three measurements were made for each spirometry and lung volume variable to ensure reproducibility and the highest value was used in subsequent calculations. The flow-sensor was calibrated before each test using a three-litre syringe.

Lung diffusion capacity for carbon monoxide (TLco) was measured by the single breath method using a mixture of carbon monoxide and methane and was measured at least in duplicate. TLC, VC, IC, FEV_1 _and TLco were expressed as a percentage of the predicted values, which were obtained from regression equations by Quanjer et al [13] and Cotes et al [14].

#### Cardiopulmonary exercise test

CPET was performed according to a standardized procedure [15]. After calibration of the oxygen and carbon dioxide analyzers and flow mass sensor, patients were asked to sit on an electromagnetically braked cycle ergometer (Corival PB, Lobe Bv, Groningen, The Netherlands) and the saddle was adjusted properly to avoid the maximal extension of the knee. The exercise protocol involved an initial 3 minutes of rest, followed by unloaded cycling for another 3 minutes with an increment every minute of 5-15 W, according to anthropometric data and functional impairment degree of the patients, in order to perform an exercise time lasting 8-12 min. Patients were asked to maintain a pedalling frequency of 60 rpm indicated by a digital display placed on the monitor of the ergometer. Breath-by-breath VO_2 _(L/min), VCO_2 _(L/min) and minute VE (L/min) were collected during the test (CPX/D; Med Graphics, St Paul, MN, U.S.A.). Patients were continuously monitored by a 12-lead electrocardiogram (Welch Allyn CardioPerfect, Delft, The Nederlands) and a pulse oximeter (Pulse Oximeter 8600, Nonin Medical Inc, MPLS, Mn U.S.A.). Blood pressure was measured at 2 min intervals. Stopping criteria consisted of symptoms such as unsustainable dyspnoea or leg fatigue, chest pain, ECG ST-segment depression, a drop in systolic blood pressure or oxygen saturation (SpO_2_) ≤ 84%.

Peak workload and peak VO_2 _were recorded as the mean value of watts and VO_2 _during the last 20 s of the test. Peak VO_2 _was expressed as absolute value in mL/kg/min and as percent of predicted value [16].

Changes in operational lung volumes were evaluated from measurements of IC at rest, every 3 min during exercise and at peak exercise. After a full explanation to each patient, satisfactory technique and reproducibility of IC manoeuvres were established during an initial practice session at rest. Assuming that TLC remains constant during exercise in COPD [17], changes in IC reflect changes in end-expiratory lung volume (EELV = TLC-IC). EELV was expressed as percent of TLC for analysis. Patients were divided in two groups according to the EELV (%TLC) at peak of exercise; the group with a EELV(%TLC) ≥ 75% was defined "heavy hyperinflators".

The cardiovascular response to exercise was expressed by the following parameters: O_2_Pulse change (ΔO_2_Pulse), DP reserve and OUES. O_2_Pulse (mL/bpm) was calculated by dividing instantaneous oxygen uptake by the heart rate [16]. ΔO_2_Pulse was calculated as follows: O_2_Pulse at maximal exercise minus O_2_Pulse at rest. DP (mmHg·bpm) at rest and at maximal exercise was calculated by the product of systolic blood pressure and heart rate [18]. DP reserve was calculated as follows: DP at maximal exercise minus DP at rest. The OUES describes the relationship between VO_2 _and VE during incremental exercise, via a log transformation of VE and was expressed as the gradient of the linear relationship of log_10 _VE to VO_2 _(L/min) [8].

#### Dyspnoea

Chronic activity-related dyspnoea was evaluated with the Italian version of a five-point MRC scale modified by the ATS [19].

Dyspnoea induced by CPET was measured at the end of the incremental exercise by a 0-100 visual analogue scale (VAS) [20]. Dyspnoea perception ratings were then divided by the maximal workload (VAS/MW in mm/watts) for analysis.

### Statistical analysis

Data are reported as mean ± standard deviation (SD), unless otherwise specified. The distribution of variables was assessed by means of Kolmogorov-Smirnov Goodness-of-Fit test. Relationships between variables were assessed by the Pearson's correlation coefficient (r) and linear regression analysis. Comparisons between variables were determined by unpaired t-test or by Chi-square test, when appropriate.

The receiver operating characteristic (ROC) curve method [21] was used to plot the true positive rate (sensitivity) in function of the false positive rate (1-specificity) for different cut-off points of ΔO_2_Pulse and DP reserve with respect to a EELV (%TLC) ≥ 75% as a threshold value.

A *p *value of less than 0.05 was taken as significant.

## Results

We screened 55 consecutive patients (18 females) with stable COPD, aged between 39 and 81 years. Seven patients did not fall within the inclusion criteria. Demographic and clinical characteristics of the 48 patients included in the study are shown in Table 1. At study entry, patients were receiving inhaled steroids (75%), long-acting beta_2_-agonists (75%) and Tiotropium (65%); all of them were ex-smokers. Overall, a wide range of airflow obstruction (FEV_1_/VC from 29 to 68%), airway collapsibility (FEF_50_/FIF_50 _ratio from 0.09 to 0.94 L/s), lung hyperinflation (IC/TLC ratio from 0.19 to 0.63 L), diffusing capacity (TLco from 38 to 105%) and chronic activity-related dyspnea (MRC from 0 to 4) were found (Table 1).

**Table 1:** Demographic, baseline and exercise characteristics of COPD patients

	All Patients (n = 48)		Patients with EELV ≥ 75% TLC (n = 23)		Patients with EELV < 75% TLC (n = 25)		
	Mean ± SD	Range	Mean ± SD	Range	Mean ± SD	Range	p*
Age (yr)	68 ± 8	39-81	70 ± 7	57-81	67 ± 10	39-81	*0.263*
Females/Males	16/32	--	7/16	--	9/16	--	*0.894*
BMI (kg/m^2^)	26 ± 4	19-36	26 ± 4	20-34	26 ± 4	19-36	*0.889*
VC (% pred)	84 ± 16	55-124	81 ± 14	55-100	87 ± 18	60-124	*0.199*
FEV_1 _(% pred)	55 ± 13	33-81	47 ± 10	33-65	62 ± 12	34-81	*0.001*
TLC (% pred)	118 ± 20	80-177	127 ± 19	88-177	111 ± 17	80-139	*0.004*
TLco (% pred)	68 ± 17	38-105	65 ± 19	38-105	72 ± 15	39-99	*0.137*
FEV_1_/VC (%)	51 ± 12	29-68	46 ± 12	29-67	56 ± 9	36-68	*0.003*
FEF_50_/FIF_50 _ratio (L/s)	0.41 ± 0.22	0.09-0.94	0.31 ± 0.18	0.09-0.88	0.49 ± 0.21	0.22-0.94	*0.003*
IC/TLC ratio (L)	0.33 ± 0.10	0.19-0.63	0.26 ± 0.04	0.19-0.36	0.40 ± 0.09	0.24-0.63	*0.001*
HR rest	81 ± 16	47-114	83 ± 17	52-114	80 ± 16	47-108	*0.486*
HR peak	118 ± 18	71-150	115 ± 21	71-147	121 ± 16	93-150	*0.299*
Sp O_2 _rest	95.6 ± 1.4	93-98	95.3 ± 1.5	93 - 98	95.8 ± 1.2	93 - 98	*0.177*
Sp O_2 _peak	94.9 ± 2.1	89-98	93.8 ± 2.3	89 - 98	95.8 ± 1.2	94 - 98	*0.0004*
Peak VO_2 _(mL/kg/min)	15.3 ± 3.2	10.6-22.5	14.1 ± 3.1	10.6-22.5	16.4 ± 2.9	11.5-21.0	*0.012*
Peak workload (watts)	75 ± 24	33-128	64 ± 22	33-106	85 ± 21	47-128	*0.001*
VAS/MW (mm/watts)	1.25 ± 0.5	0.51-2.94	1.07 ± 0.3	0.63-1.76	1.44 ± 0.56	0.51-2.94	*0.006*
ΔO_2_Pulse (mL/bpm)	9.63 ± 2.68	5.0-15.0	8.65 ± 2.21	5.0-15.0	10.52 ± 2.80	6.0-15.0	*0.014*
DP reserve (mm Hg · bpm)	10,431 ± 4,165	1,460-20,560	8,509 ± 3,537	1,460-16,740	12,074 ± 3,862	4,360-20,560	*0.017*

*p value: patients with EELV ≥ 75% TLC vs patients with EELV < 75% TLC

Twenty-five (8 females) out of 48 patients (52%) suffered from arterial hypertension and were on diuretics (72%), ACE-inhibitors (52%), Ca-antagonists (36%), beta-blockers (28%) to control the disease.

All patients completed the exercise test; at the peak workload was 75 watt ± 24 (range 33-128 watt) without any complication. Mean peak VO_2 _values were 15.3 ± 3.2 (range 10.6-22.5) and 63 ± 16 (range 33-112), when expressed respectively in mL/kg/min and as percent of predicted value. Mean VAS/MW was 1.25 mm/watts ± 0.48 (range 0.51-2.94). There was a statistically significant fall in the SpO_2_, from 95.6% ± 1.4 (range 93-98%) to 94.9% ± 2.1 (range 89-98%) (*p = 0.007*). Mean resting IC and EELV (%TLC) were 2.27 L ± 0.69 (82 ± 19% of predicted) and 66.5% ± 9.5, and significantly changed by +0.38 L ± 0.41 (range -1.36 to 0.53 L; *p < 0.0001*) and -7.88% ± 8.51 (range -7.90 to 30.4%; *p < 0.0001*), respectively.

EELV (%TLC) at peak exercise was 72% ± 11, being ≥ 75% in 23 out of 48 patients. Patients with a peak exercise EELV (%TLC) ≥ 75% showed significantly lower FEV_1_/VC, FEF_50_/FIF_50 _ratio and IC/TLC ratio compared with the others (Table 1).

When related to the resting lung function, EELV (%TLC) showed a negative correlation with IC/TLC (*r = - 0.871, p < 0.0001*), FEV_1_/VC (*r = - 0.435, p = 0.002*), and FEF_50_/FIF_50 _(*r = - 0.391, p = 0.006*). Moreover, it was significantly and inversely related to peak workload (*r = - 0.540, p < 0.0001*) and peak VO_2 _(*r = - 0.452, p = 0.001*) and directly related to VAS/MW (*r = 0.501, p = 0.0002*).

At baseline and at peak of exercise, mean O_2_Pulse and DP were 3.69 mL/bpm ± 1.36 and 9.63 mL/bpm ± 2.68 and 10,746 mm Hg · bpm ± 2,664 and 21,006 mmHg · bpm ± 5,154, respectively. ΔO_2_Pulse, DP reserve and OUES were 5.94 mL/bpm ± 2.03, 10,431 mmHg · bpm ± 4,165, and 1.39 L/min ± 0.33, respectively. Patients with a peak exercise EELV (%TLC) ≥ 75% showed significantly lower ΔO_2_Pulse, OUES and DP reserve values compared to others (Table 1). Furthermore, SpO_2 _values at peak exercise were significantly lower in the heavy hyperinflators, as compared to those of the remaining patients (Table 1).

**Figure 1:**
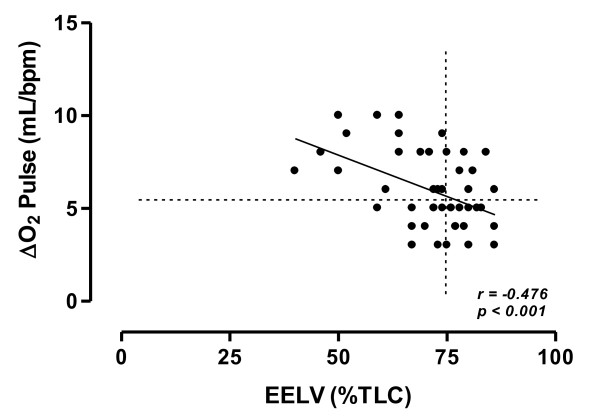
**Relationship between oxygen pulse change from rest to peak exercise (ΔO_2 _Pulse) and EELV (%TLC) at peak of exercise in the study population**. Horizontal and vertical interrupted lines correspond to 5.5 mL/bpm ΔO_2 _Pulse value and to 75% EELV of TLC value, respectively. According to the ROC curve method, a ΔO_2 _Pulse ≤ 5.5 mL/bpm was the cut-off point, which maximized sensitivity and specificity (0.640 and 0.696, respectively) with respect to EELV (% TLC) ≥ 75%, as a threshold value. Continuous line is the linear regression line.

**Figure 2:**
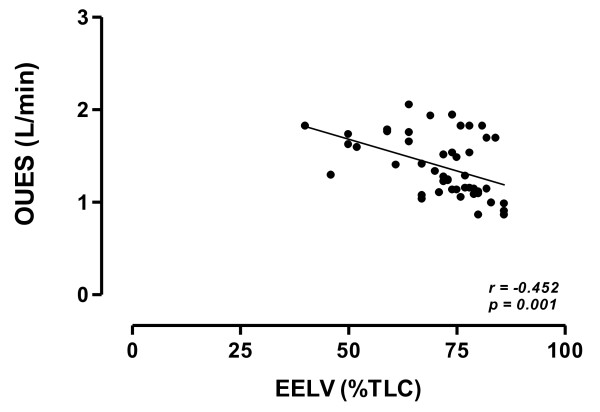
**Relationship between oxygen uptake efficiency slope (OUES) during exercise and EELV (TLC) at peak of exercise in the study population**. Continuous line is the linear regression line.

**Figure 3:**
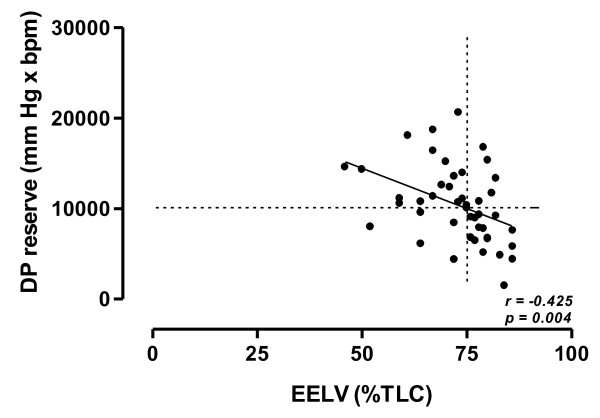
**Relationship between double product change from rest to peak exercise (DP reserve) and EELV (%TLC) at peak of exercise in the study population**. Horizontal and vertical interrupted lines correspond to 10,000 mmHg · bpm DP reserve value and to 75% EELV of TLC value, respectively. According to the ROC curve method, a DP reserve ≤ 10,000 mmHg · bpm was the cut-off point, which maximized sensitivity and specificity (0.720 and 0.783, respectively) with respect to EELV (% TLC) ≥ 75%, as a threshold value. Continuous line is the linear regression line.

EELV (%TLC) showed a negative correlation with ΔO_2_Pulse (*r = - 0.476, p = 0.001*) (Figure 1), OUES (*r = - 0.452, p = 0.001*) (Figure 2) and DP reserve (*r = - 0.425, p = 0.004*) (Figure 3).

According to the ROC curve method, the plot of the true positive rate in function of the false positive rate for different cut-off points of ΔO_2_Pulse change and DP reserve with respect to EELV (% TLC) ≥ 75% as a threshold value showed respectively 0.703 (*p = 0.016*) and 0.767 (*p = 0.002*) area under curve value. The ΔO_2_Pulse change and DP reserve cut-off points, which maximized sensitivity and specificity, were ≤ 5.5 mL/bpm (0.640 sensitivity and 0.696 specificity) and ≤ 10,000 mmHg · bpm (0.720 sensitivity and 0.783 specificity), respectively.

**Figure 4:**
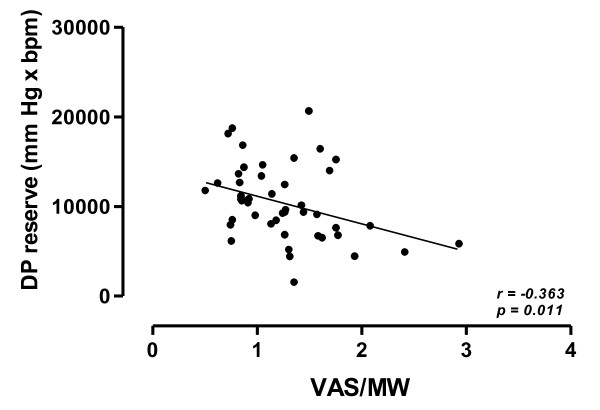
**Relationship between breathlessness perception, as corrected for workload in watts, at peak of exercise (VAS/MW) and DP reserve values in the study population**. Continuous line is the linear regression line.

In addition, in patients with a DP reserve ≤ 10,000 mmHg · bpm, as compared to those of patients with DP reserve > 10,000, VAS/MW values were significantly higher (1.471 mm/watts ± 0.52 *vs *1.045 mm/watts ± 0.34; *p = 0.001*). In all patients, VAS/MW values were significantly and negatively related to DP reserve values (*r = -0.414; p = 0.008*) (Figure 4).

In all patients, a significant correlation between O_2_Pulse at peak exercise (*r = 0.481, p = 0.0006*), ΔO_2_Pulse (*r = 0.458, p = 0.001*), DP reserve (*r = 0.295, p = 0.046*), OUES (*r = 0.404, p = 0.005*) and resting IC/TLC was found.

Hypertensive patients did not differ from normotensive patients both in terms of resting and exercise values, including the ΔO_2_Pulse, DP and OUES.

## Discussion

The main finding of the present study is that dynamic hyperinflation is strictly associated to a poor cardiovascular response to exercise in COPD patients with different degree of severity. This finding supports the view that in COPD patients, dynamic hyperinflation may affect exercise performance not only by impairing ventilation, but also cardiac function. In addition, this study confirmed that dynamic hyperinflation is related to baseline lung function and to a poor exercise performance, both in terms of workload and oxygen uptake.

In larger population studies, it has been shown that more than 80% of COPD patients had a significant decrease in IC during incremental work rate cycle exercise [1,22] which varied inversely with the level of resting hyperinflation [22]. We have now confirmed and extended that finding by providing the evidence that dynamic hyperinflation, as assessed by EELV at peak of exercise, was inversely related to resting hyperinflation and to airway obstruction and collapsibility. It is of note that, in COPD patients, airway collapsibility is due to the calibre of intraparenchymal airways which is much less on expiration than on inspiration because of the lack of parenchymal support in the peripheral airways, as a consequence of disruption of the elastic network of the lung. Airway collapsibility, indeed, expressed as the FEF_50_/FIF_50 _ratio, was found significantly and inversely related to the extent of pulmonary emphysema, as assessed by means of lung scan [23].

In line with previous studies [22,24], we found that dynamic hyperinflation was inversely related to the extent of exercise capacity. Interestingly, we also demonstrated that in COPD patients dynamic hyperinflation was associated with a poor cardiovascular response to exercise. Previously, in a large cohort of COPD patients, a strong positive correlation between resting IC/TLC ratio and the left ventricular end-diastolic diameter was found [25]. Butler et al [26] showed that in COPD patients exercising in supine position, the raised wedge pressure was partly due to a rise in pressure in the cardiac fossa associated with lower lobe gas trapping. It is of note that left ventricular dysfunction, echocardiographically measured, was associated with a reduced physical activity in COPD patients [27].

Recently, in a cohort of patients with severe COPD Vassaux et al [5] found that both resting and peak exercise IC/TLC ratios were associated with oxygen pulse. In particular, patients with resting IC/TLC ≤ 25% had lower peak oxygen pulse, as compared to patients with IC/TLC > 25%. In addition, a significant and inverse relationship was found between changes in IC and oxygen pulse from rest to peak exercise. In the present study, we confirmed this finding in COPD patients with a wide range of airflow obstruction, showing a significant and inverse relationship between the peak exercise EELV and the change in oxygen pulse achieved on exercise. Moreover, the change in oxygen pulse during exercise was lower in heavy hyperinflators than in the remaining patients.

The oxygen pulse does not directly measure the stroke volume, but it may be considered as a surrogate marker, when arterial oxygen content can be assumed to be normal [6]. Accordingly, in normal subjects, exercise stroke volume may be estimated simply as five times the slope of the linear oxygen uptake-heart rate relationship [28]. Our study was non-invasively performed and, thus, we did not measure the true arterial oxygen content in our patients. Physiologically, the arterial oxygen content can be affected by the availability of haemoglobin, blood oxygenation in the lung and oxygen extraction in the periphery. Notwithstanding, we excluded patients with concomitant anaemia in our study, whereas the resting oxygen saturation of patients was quite normal and its change at peak exercise was statistically, but not clinically, significant. In COPD patients, the oxygen extraction during exercise can be similar [29] or lower [30] as compared to controls, however these differences cannot account for the decrease in oxygen pulse, since a lower oxygen extraction would actually magnify the oxygen pulse value.

Dynamic hyperinflation may reduce left ventricle stroke volume since increases intrathoracic pressures and, consequently, decreases the preload by reducing both venous return and the volume of the left ventricle. As a consequence, there is a reduced filling of the left ventricle and consequently a reduced cardiac output. Interestingly, in very severe COPD patients, Montes de Oca et al [31] found a direct relationship between inspiratory intrathoracic pressures and oxygen pulse at peak of exercise. In our study, we had a further evidence of the effect of dynamic hyperinflation on left ventricle stroke volume. Indeed and most important, we found that DP reserve was inversely related to the dynamic hyperinflation during exercise. In addition, heavy hyperinflators had a high likelihood to have a DP reserve value ≤ 10,000 mmHg · bpm. Lastly, in all patients, DP reserve was inversely related to breathlessness perception at peak exercise, suggesting that in COPD patients the exertional dyspnoea may be due not only to ventilatory constrains, but also to a functional cardiovascular impairment.

DP reserve is used as an estimate of the maximal performance of the left ventricle. It would appear that the capacity to achieve an adequate systolic blood pressure requires a relatively normal functioning left ventricle such that cardiac output increases in proportion to the increase in work rate and that it can be sustained even when exertion is near maximal [7]. DP reserve reflects myocardial oxygen uptake during exercise, since the three major determinants of myocardial oxygen uptake are the tension in the wall of the ventricle, the contractile state of the heart and the heart rate [7]. Notably, studies in healthy subjects [32] and in patients with angina pectoris [18] showed that the myocardial oxygen consumption during exercise can be reliably estimated by the DP value. Interestingly, a DP reserve ≤ 10,000 mmHg · bpm is considered as a predictor of cardiovascular mortality [7].

Finally, we also found that OUES was negatively associated with hyperinflation at peak of exercise. The OUES is the rate of increase of VO_2 _in response to a given VE during incremental exercise [33]. OUES indicates how effectively oxygen is extracted and taken into the body and in essence represents the absolute rate of increase in VO_2 _per 10-fold increase in ventilation. OUES is a parameter that integrates the functional capacities of several organ systems (primarily cardiovascular, musculoskeletal and pulmonary) during exercise, since it can be affected by the lactic acidosis onset, muscle mass, oxygen extraction and utilization, and the physiologic pulmonary dead space ventilation. In the present study, we found that for any given amount of ventilation, the higher was hyperinflation the lower was oxygen uptake during exercise (Figure 2). Therefore this finding suggests that a reduced cardiovascular fitness is associated to dynamic hyperinflation in COPD patients, although we cannot exclude that some degree of deconditioning may also affect the relationship between oxygen uptake and ventilation during exercise in the most hyperinflated patients.

## Conclusions

In conclusion, the present study clearly shows that COPD patients with dynamic hyperinflation have a poor cardiovascular response to exercise. Although association does not imply causality, our findings suggest that ventilatory constraints during exercise may play a relevant role on cardiovascular function in patients with COPD. These findings may also have some direct clinical implication. In fact, it is likely that during rehabilitation hyperinflated COPD patients might differently behave and response to exercise, thus, having a different impact on their disability and symptoms in the long-term and it should be a field for further investigations.

## Abbreviations

ACE: angiotensin converting enzyme; BMI: body mass index; COPD: chronic obstructive pulmonary disease; CPET: cardiopulmonary exercise test; DP: double product; EELV: end-expiratory lung volume; FEV_1_: forced expiratory volume in 1^st ^second; FEF_50_: forced expiratory flow at 50% of FVC; FIF_50_: forced inspiratory flow at 50% of FVC; FVC: forced vital capacity; HR: heart rate; IC: inspiratory capacity; MW: maximal workload; O_2_pulse: oxygen pulse; OUES: oxygen uptake efficiency slope; RER: respiratory exchange ratio; ROC: receiver operating characteristic; SpO_2_: oxygen saturation; SD: standard deviation; TGV: thoracic gas volume; TLC: total lung capacity; TLco: lung diffusion capacity for carbon monoxide; VAS: visual analogue scale; VC: vital capacity; VCO_2_: carbon dioxide production; VE: ventilation; VO_2_: oxygen uptake.

## Conflict of interests

The authors declare that they have no competing interests.

## Authors' contributions

PT served as the primary author. She developed the study protocol, participated in the patients recruitment and statistical analysis and drafted the manuscript and she is the guarantor of the entire manuscript. MA participated in the design of the study and helped to patients recruitment. DE and LB participated in the design of the study and the statistical analysis and helped to draft the manuscript. EM and DO participated in the coordination of the study. EC participated in the design of the study and helped to draft the manuscript. AC developed the study protocol, interpreted study data, developed the first draft of the manuscript, contributed to and reviewed drafts of the manuscript. All authors read and approved the final manuscript.
